# Machine Learning Assisted Clustering of Nanoparticle Structures

**DOI:** 10.1021/acs.jcim.2c01203

**Published:** 2023-01-04

**Authors:** Cesare Roncaglia, Riccardo Ferrando

**Affiliations:** † Physics Department, University of Genoa, Via Dodecaneso 33, 16146Genoa, Italy; ‡ Physics Department, University of Genoa and CNR-IMEM, Via Dodecaneso 33, 16146Genoa, Italy

## Abstract

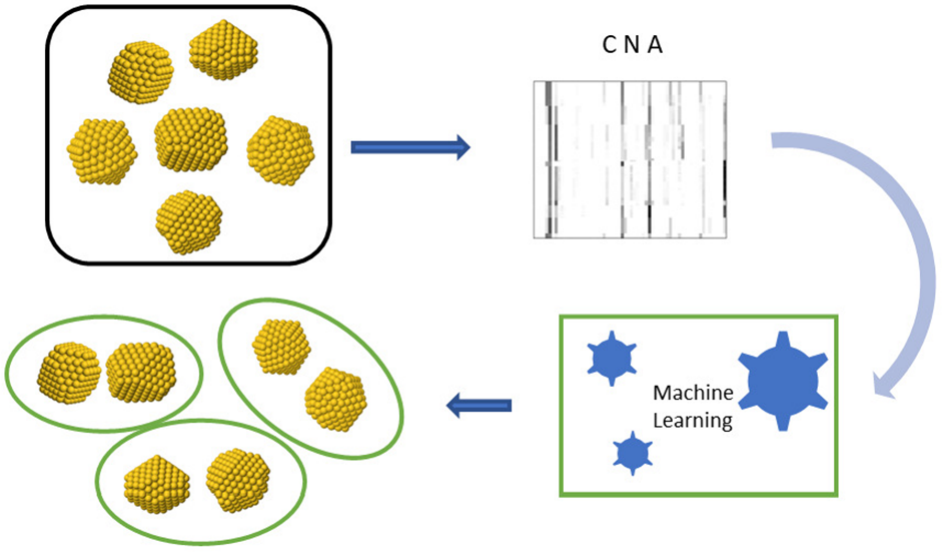

We propose a scheme for the automatic separation (i.e., clustering) of data sets composed of several nanoparticle (NP) structures by means of Machine Learning techniques. These data sets originate from atomistic simulations, such as global optimizations searches and molecular dynamics simulations, which can produce large outputs that are often difficult to inspect by hand. By combining a description of NPs based on their local atomic environment with unsupervised learning algorithms, such as K-Means and Gaussian mixture model, we are able to distinguish between different structural motifs (e.g., icosahedra, decahedra, polyicosahedra, fcc fragments, twins, and so on). We show that this method is able to improve over the results obtained previously thanks to the successful implementation of a more detailed description of NPs, especially for systems showing a large variety of structures, including disordered ones.

## Introduction

Metal nanoparticles (NPs) are aggregates of atoms at the nanoscale. Due to their finite nature, they differ considerably from their well-known bulk counterparts. For this reason, in the last decades, these interesting objects have received a lot of attention both from experimental and theoretical research.^1^ Such an effort shed some light on structural, physical, and chemical properties that suggested a large variety of real-world applications, such as catalysis,^2,3^ biomedicine,^4^ plasmonics,^5^ and many more. However, due to all the difficulties that arise from either experimental preparation, observation and theoretical modeling, there are still plenty of mysteries to solve. In this scenario, computer simulations established a promising bridge between theories and experiments, by showing an incredible capability of solving some of those problems otherwise too hard to tackle.^6^ With resources nowadays, for example, some properties of a nanoparticle composed of some hundreds of atoms can be studied *ab initio* by means of Density Functional Theory (DFT) in a reasonable amount of time. Larger sizes and time scales can be studied by simplified atomistic approaches, which do not take into account explicitly the electrons of a system, thus resulting in much faster calculations. Typically, the optimal structure (and also the chemical ordering if there are two or more metals) of an NP is searched by global optimizations techniques, such as basin hopping (BH) or genetic algorithm, which implement clever ideas to explore the atomistic potential energy landscape. Then, thanks to Molecular Dynamics (MD) simulations, the thermal evolution and/or growth of an NP composed of thousands of atoms can be followed for tens of μs.^7^ Even larger sizes and time scales are achieved by coarse-grained models, in which also complex environments of nanoparticles (for example, solid substrates and embedding matrices) can be simulated and studied.^8^

A common feature of these simulations is the production of large outputs, usually many NP structures in some three-dimensional coordinates file format. The systematic analysis of all these structures can be far from a trivial task for complex systems, and since these data usually contain a crucial amount of information, this procedure cannot be simply bypassed. Automatic tools capable of doing this job are then quite convenient.

Here, we propose a procedure based on a combined approach of Machine Learning and the Common Neighbor Analysis (CNA). We show that the latter is very well suited for a successful and physically meaningful description of NPs that is necessary for unsupervised learning algorithms to solve the clustering problem efficiently. More precisely, we show that the best results are obtained thanks to the highly detailed description of the local environment of atoms that we give, similar to some works recently published.^9,10^ We also show and stress the importance of Machine Learning in the whole process. This comes essentially in two steps: the first one is the extraction of the most relevant information from the above-mentioned complex description through a dimensionality reduction technique, which is achieved by Principal Component Analysis (PCA), and the second one is the combined choice of a clustering algorithm and of a clustering score, which is needed in order to select the best output. This choice was made out of a combination of different algorithms (K-Means and Gaussian Mixture Model) and scores (Bayesian criterion information, silhouette, and gap statistic). More details will be given below in the next section. Thanks to the more refined approach used here, we also demonstrate that it is possible to improve the performance of the method introduced in refs (11) and (12). We believe that our latest method can be used as an effective recipe to more easily study all the different geometric structures that emerge from computer simulations of metal nanoparticles, allowing the user to quickly grasp some subtle differences between seemingly similar objects. The method will be applied to the following systems: AgCu, AuPd, Au, and Ag nanoparticles. These systems have been widely studied in the literature so far, both theoretically and experimentally, for their applications to catalysis^13,14^ and plasmonics.^15,16^

A note on terminology: In order to avoid confusion, aggregates of atoms will be referred to as *nanoparticles* or *nanoalloys*. On the other hand, the term *cluster* will be used exclusively to denote a set of nanoparticle structures which are grouped together by a clustering algorithm working in the space of suitable variables (i.e., of structural descriptors).

## Models and Methods

### Data Collection and Representation

Nanoparticle structures were collected by means of global optimizations and molecular dynamics simulations. A set of nanoparticle structures given by three-dimensional atomic positions makes a data set. For all the systems we analyzed, atomic interactions were modeled by a Gupta potential that can be derived as an approximation to the tight binding model^17,18^ (see the SI for details).

The parameters of the potentials were taken from ref (19) for Ag and AgCu, from ref (20) for Au, and from ref (21) for AuPd. Data about the accuracy of these model potentials in comparison with experiments and Density Functional Theory calculations can be found in refs (11), (22), and (23).

Global optimization searches were made by the Basin Hopping algorithm,^24^ using our own code.^25,26^

For constant-temperature molecular dynamics simulations, the equations of motions derived from the above-mentioned Gupta potential were solved using the Verlet algorithm^27^ coupled with the Andersen thermostat,^28^ using our own code.^29^ For each MD simulation, the temperature of the thermostat was chosen in the solid–liquid transition range. This choice allows a sampling of different geometrical configurations of isomers, since nanoparticles are more likely to fluctuate at this temperature. On the contrary, a low temperature would in fact trap the system in the nearest local minimum and there would not be crossing of barriers, and the data set would be composed of mostly identical structures.

Once all NP structures were collected, they were described in terms of the Common Neighbor Analysis (CNA).^30^ In particular, the CNA assigns to each pair of nearest neighbors a signature of three integers *rst* that depends on their local environment:

- *r* - The number of common nearest neighbors of the pair.
- *s* - The number of bonds between those *r* atoms, i.e. the number of nearest neighbor pairs in these *r* atoms.
- *t* - The length of the longest chain of bonds that can be made out of the *s* bonds present.

**Figure 1:**
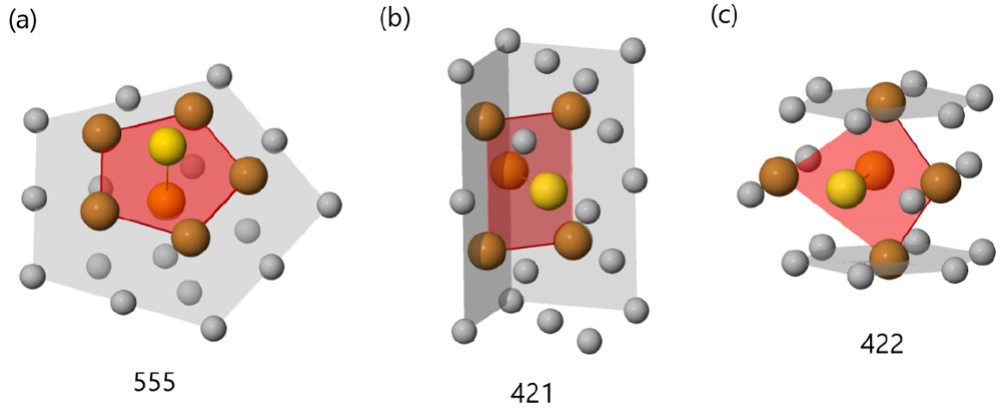
Three typical CNA signatures: (a) a 555 signature, which denotes atom pairs along 5-fold symmetry axes, such as those of icosahedra and decahedra, (b) a 421 signature, which is that of a perfect FCC crystal fragment, and (c) a 422 signature, which is instead typical of twin planes, fcc stacking faults, and hcp stackings. In all cases, yellow atoms are the reference atom pair for which one investigates the neighborhood, whereas the common nearest neighbors of the pair (corresponding to the integer *r*) are colored in brown. Bonds (corresponding to the integer *s*) are represented by segments between brown atoms.

As an example, consider the atoms in a 5-fold symmetry axis. A pair of nearest neighbors lying on that axis will have five common nearest neighbors placed on a pentagon, all of which are nearest neighbors. Thus, the pair has a 555 signature (see Figure 1(a)).

**Figure 2:**
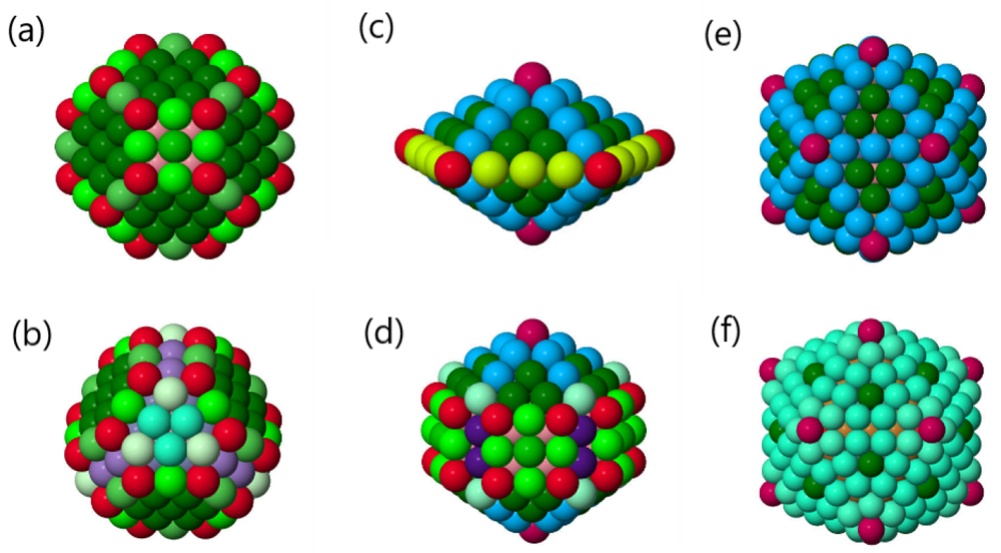
(a) Regular truncated octahedron, (b) truncated tetrahedron with islands in stacking faults on its facets, (c) regular decahedron, (d) Marks decahedron, (e) Mackay icosahedron, and (f) anti-Mackay icosahedron. Different colors refer to different atom classifications (see Table 1).

In principle, each atom can be classified with the signatures calculated with all the pairs formed with all its nearest neighbors. However, this would open up too many possibilities, so that we restricted ourselves only to a limited number of possible combinations. In fact, the analysis of a single data set composed of several nanoparticle structures will deliver a huge amount of different possible combinations of signatures, especially for the data sets collected in molecular dynamics simulations. Indeed, we checked that for some of our data sets there are thousands of these possible atom classifications. However, most of these combinations are rarely explored along the MD trajectory. Very often, a particular classification is explored by just one atom in a single structure for the whole simulation. In such a case, it can be discarded as its frequency is very low - and the corresponding geometry barely relevant with respect to other, more frequent features. Note that some of these combinations appear much more often than others, as they represent atoms in well-defined positions. For example, an atom in a perfect fcc crystal will have 12 nearest neighbors, all of which form a 421 signature (see Figure 1(b)). Thus, this kind of atom can be classified by a list of 12 421 signatures. An atom placed in the inner part of a 5-fold axis has 12 nearest neighbors. Two of these neighbors, the ones placed in the 5-fold axis, just above and below it, form a 555 signature, with the original atom, while the remaining ten yield a 422 signature. A vertex of an icosahedron or a vertex of a decahedron has six nearest neighbors, five of which make a 321 signature, whereas with the last one, placed just below in the 5-fold axis, forms a 555 signature. In Figure 2, a selection of structures is provided with atoms colored differently to enhance their various classifications.

We defined a list of 63 different combinations of signatures; the complete list of atom classifications can be found in Table S1 in the SI. Following this list, each NP of each simulation was then assigned a 64-dimensional vector, in which for the *i*-th component the percentage of atoms of that NP having the *i*-th combination of signatures was calculated. This list of 63 different combinations then comes from the trade-off for a detailed but contained range of possible atom classifications. The last component of the vector was dedicated to all the atoms that were not classified, that is those atoms whose local environment could not be described in terms of one of the 63 combinations of the list (This percentage of not-classified atoms reflected our decision to select these 63 combinations, and it would be zero in the extreme case of considering all the possible combinations that appeared in each NP in a particular data set.). Some of the notable possible combinations of signatures are listed in Table 1.

**Table 1:**
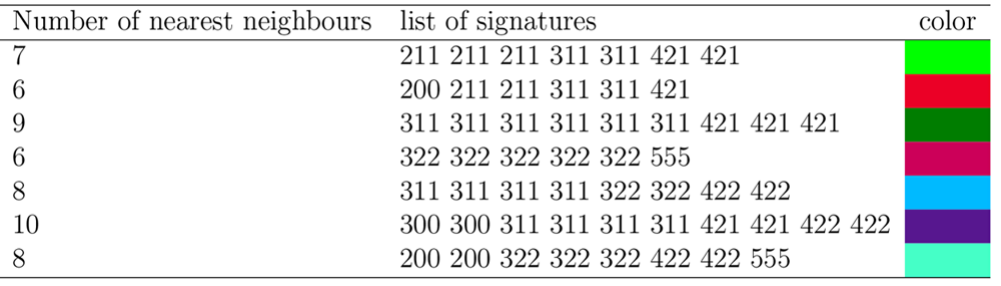
Some Atom Classifications Based on the List of Signatures of Its Nearest Neighbors, Taken as Examples from Figure 2^a^

a The complete list of atom classifications can be found in Table S1 in the SI.

**Figure 3:**
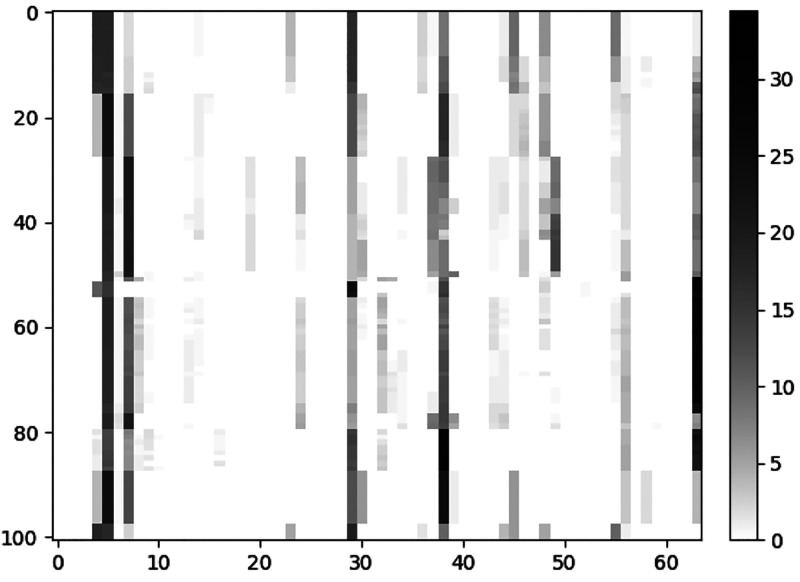
Example of the 64-dimensional vector representation of nanoparticles given by the CNA. In this case, each row represents a AgCu nanoalloy with a different composition of Ag and Cu; each column represents a vector component whose values (i.e., percentages) are indicated in the color bar. Data are from ref (11).

Each data set was then described by a collection of 64-dimensional vectors, that is an *n* × 64 matrix, where *n* is the number of structures of the data set. A visualization of one of these matrices is given for example in Figure 3, where for each row a vector (i.e., an NP structure) is represented by coloring each component (i.e., column) in a reverse grayscale mode. This type of visualization allows to detect, at a first glance, the most frequent signatures (i.e., the darker components) in the whole data set, which also serves as a rough first estimate of its variation.

### Machine Learning

Before any application of clustering algorithms on the 64-dimensional space, we performed PCA^31^ in order to reduce the dimensionality of our data. This technique is one of the most popular procedures in the general problem of dimensionality reduction, i.e. the problem of searching for a map which transforms a high-dimensional data set into a simpler, low-dimensional version of it, still preserving some of its properties. In particular, the aim of PCA is to look for a set of orthonormal vectors (i.e., directions) in the orginal space along which the variance of the data set is maximum. In other words, if the original data set consists of *n* different *d*-dimensional vectors *x*_*i*_, the first principal component *w*_1_^*^ is defined as the solution of the following maximization problem
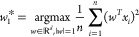
1that is, *w*_1_^*^ is the direction along which the variance of the data is maximum, with *w* denoting an arbitrary direction in the *d*-dimensional space. The second principal component is instead calculated by first taking the residuals of the data set,  (that is by subtracting the “first” variance), and then looking for another *d*-dimensional unitary vector, orthogonal to *w*_1_^*^, along which the variance of the residuals is maximum. The procedure for the other components is similar, and it can be reiterated for a number of *p* components, usually with *p* ≪ *d*. Finally, the data set is transformed by taking all the projections along the principal components , where *w*_i_^*^ is the *p* principal components. The algorithm has a time complexity of , where *n*_*max*_ = max(*n*, *d*), or , where *n*_*min*_ = min(*n*, *d*), depending on the data set. The algorithm for PCA is implemented via the open source python module scikit-learn.^32^ In this module, the singular value decomposition that is needed for PCA is calculated differently depending on the shape of the input data and the number of components to extract, hence the different time complexities. More details can be found in ref (33).

We discovered that in most cases a reduction to a three-dimensional space given by the first three principal components is sufficient to keep at least 90–95% of the entire variance of the data set. This percentage defines the trade-off between a simple description (i.e., number of principal components, and so the number of dimensions to describe the data set) and precision (amount of variance). A larger number of components will give a larger percentage of variance explained; but it will not benefit the possibility of a simple visualization and also (and maybe more importantly) it will be heavier for clustering calculations, as it will be shown below by time complexities. Although there is no theoretical recipe (at least to our knowledge) for choosing a particular percentage, this number (90–95%) is a commonly accepted threshold.^34,35^ Moreover, from our experience, the addition of extra dimensions produces a little gain in variance, so that a two- or three-dimensional space is much more preferable for it is easier to represent. Each data set was then transformed from an *n* × 64 matrix to an *n* × 3 matrix (the only exception being the very first data set that will be analyzed, for which it was necessary to add a fourth component to retain at least 90% of the variance).

The reason for operating such a transformation before the clustering approach is the following. If, hypothetically, some groups of different structural families (i.e., clusters) do exist in a data set, then it is reasonable to assume that these different groups shall be somehow separated and distinguished in their original space. By applying PCA, we look for a simpler low-dimensional description in the subspace spanned by the chosen principal components, so that a good amount of variance is still present. Thus, if this variance is representative of distances between points (and so between clusters, to some extent), we can assume to preserve this separation between clusters also in the lower-dimensional space given by the principal component analysis. In our case, the goal of this procedure is then to embed the clusters of our data sets in simpler spaces by preserving some of the information contained in the 64-dimensional description given by the CNA.

In order to perform clustering on these reduced data sets, we used K-Means^36^ and GMM.^37−39^ The first is a geometrical approach, that works as follows. Let *x*_1_, *x*_2_, ..., *x*_*n*_ be *n* vectors (in *d* dimensions) to cluster and let *k* be the number of clusters, fixed a priori. The aim of this algorithm is to find the Voronoi partition of the space formed by *k* Voronoi cells assigned to *k* “mean” vectors *m*_1_^*^, *m*_2_^*^, ..., *m*_k_^*^. The partition is then given by the solution of the following problem
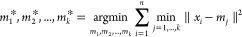
2which is typically solved by an Expectation-Maximization (EM) iterative algorithm, such as the one described in ref (36). The time complexity is given by *O*(*tknd*), where *t* is the number of iterations.

The second is instead a probabilistic approach. This model assumes that *x*_1_, *x*_2_, ..., *x*_*n*_ have been generated according to some *k* Gaussian distributions with unknown parameters (including weights, means, and covariance matrices of all *k* distributions). These parameters are estimated after the maximization of the log-likelihood of the mixture model, that is
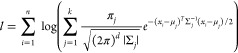
3where π_*j*_ are models weights, Σ_*j*_ are covariance matrices, and μ_*j*_ are Gaussian distributions centers, with *d* being the dimension of *x*_*i*_ vectors. The maximization is carried out by an EM algorithm, see for example refs (37), (38), and (39). The time complexity is given by *O*(*tknd*^3^), where again *t* is the number of iterations.

The optimal number of clusters was estimated by looking at the Bayesian information criterion (Bic) score,^40^ the silhouette score,^41,42^ and the gap statistic.^43^ The Bic score can be used for GMM, and it is defined as
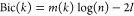
4where *m*(*k*) is the total number of parameters of the mixture model with *k* components, and  is the log-likelihood of the model as given in eq 3. The optimal number of clusters is the number *k* for which the Bic score is minimized. The silhouette score of a clustered data set is instead defined as the average of silhouette coefficients *s*_*i*_ of each point *x*_*i*_
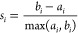
5where *a*_*i*_ is the average distance from *x*_*i*_ to other points in its cluster, whereas *b*_*i*_ is the smallest average distance from *x*_*i*_ to other instances of other clusters. In this case, the optimal number of clusters is the one that maximizes the silhouette score.

Finally, the gap statistic is defined as follows. Suppose we have *n* points in the data set divided into *k* clusters labeled *C*_1_, *C*_2_, ..., *C*_*k*_ each with *n*_1_, *n*_2_, ..., *n*_*k*_ elements in it. Suppose we take *d*_*ij*_ to be some distance between *x*_*i*_ and *x*_*j*_ in the data set; let
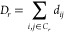
6be the sum of distances in the *r*-th cluster, which measures the dispersion in that particular cluster and let
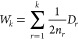
7be the overall dispersion in the data set, for a given *k*. The quantity
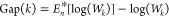
8is then used to calculate the gap statistic score, where the expectation is taken under a null reference distribution of the data. The optimal number of clusters is selected using the “1-standard error” rule, that is we look for the smallest *k* which satisfies
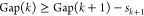
9where *s*_*k*+1_ is an error calculated from the values of log(*W*_*k*_) used for the approximation of the expectation (more details can be found in ref (43)).

Other scores have been proposed in the literature, such as Caliński-Harabasz,^44^ Krzanowski-Lai,^45^ and Hartigan.^46^ However, they have not been considered here since it was shown in ref (43) that the gap statistic usually outperforms them.

**Figure 4:**
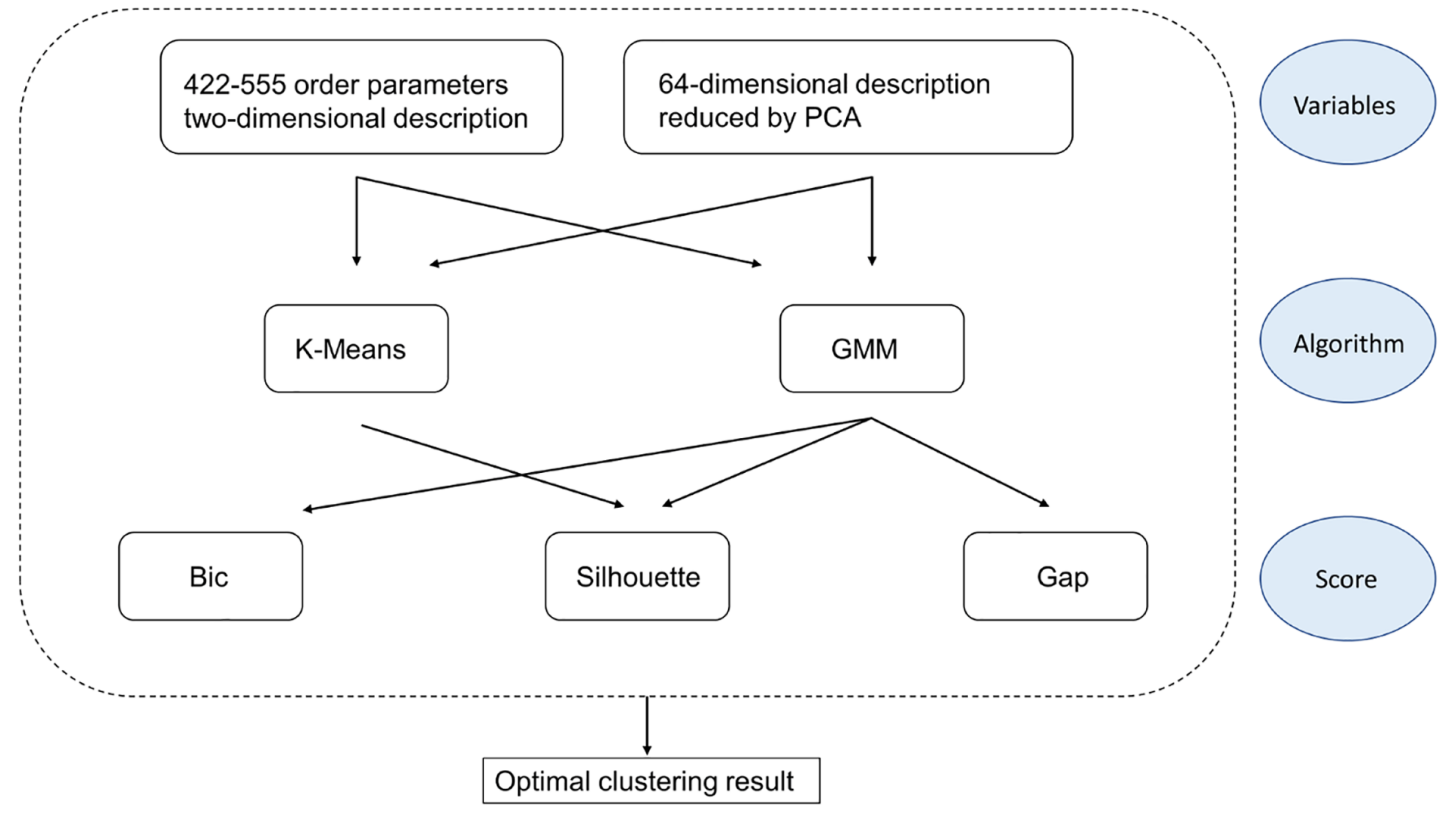
Workflow scheme for the Machine Learning analysis.

Machine Learning algorithms were implemented thanks to the open source python module scikit-learn.^32^ A schematic representation of the workflow for the Machine Learning analysis is depicted in Figure 4, where in the first row (variables) also the two-dimensional description given by two CNA signatures 422 and 555 has been added, since it was used as a descriptor for NPs in our previous works,^11,12^ and it will be used also here for comparison. Discussion about these choices can be found in the next section dedicated to results.

## Results

In this section, we provide the results for all the different systems we analyzed, highlighting differences and improvements with respect to the results obtained following the scheme already established in our previous works,^11,12^ where nanoparticles were described in terms of a two-dimensional space given by the order parameters that can be derived from the 422 and 555 CNA signatures. In particular, these two order parameters are calculated by taking the fraction of nearest-neighbors pairs of atoms having these two *rst* integers (as explained in the subsection “Data Collection and Representation”) out of all nearest-neighbors pairs. Here in this work, we also considered this two-dimensional description for a direct comparison. Since it is known that the iterative EM algorithm used for solving problems (2, 3) reaches a local optimum, we performed for each data set different independent calculations in order to better monitor this effect. The scheme is the following: first, a range for the number of cluster *k* was chosen, then for each *k* the model (GMM or K-Means) was fitted using 50 random initializations, the best result out of these 50 was kept (this is automatically done by scikit-learn), and the score (silhouette, Bic, or gap) was calculated. The best value for the score in the range was then saved. The whole operation was repeated for a total of 50 times, in order to mitigate the local optimum effect. Finally, the optimal number of clusters was deduced by looking at the most frequently chosen best score in the related histogram.

It may happen in some cases that the scores have more than one local optimum. Here, we restricted ourselves by considering only the highest or lowest values for the silhouette and Bic score respectively, since the study of other suboptimal scores for each system that we analyzed would result in a way too large for discussion. However, we would like to encourage anyone trying to use this scheme, to take a look also at those results arising from other local optima, especially when unsatisfied with what is already obtained.

### AgCu, *N* = 100 and *N* = 200

**Figure 5:**
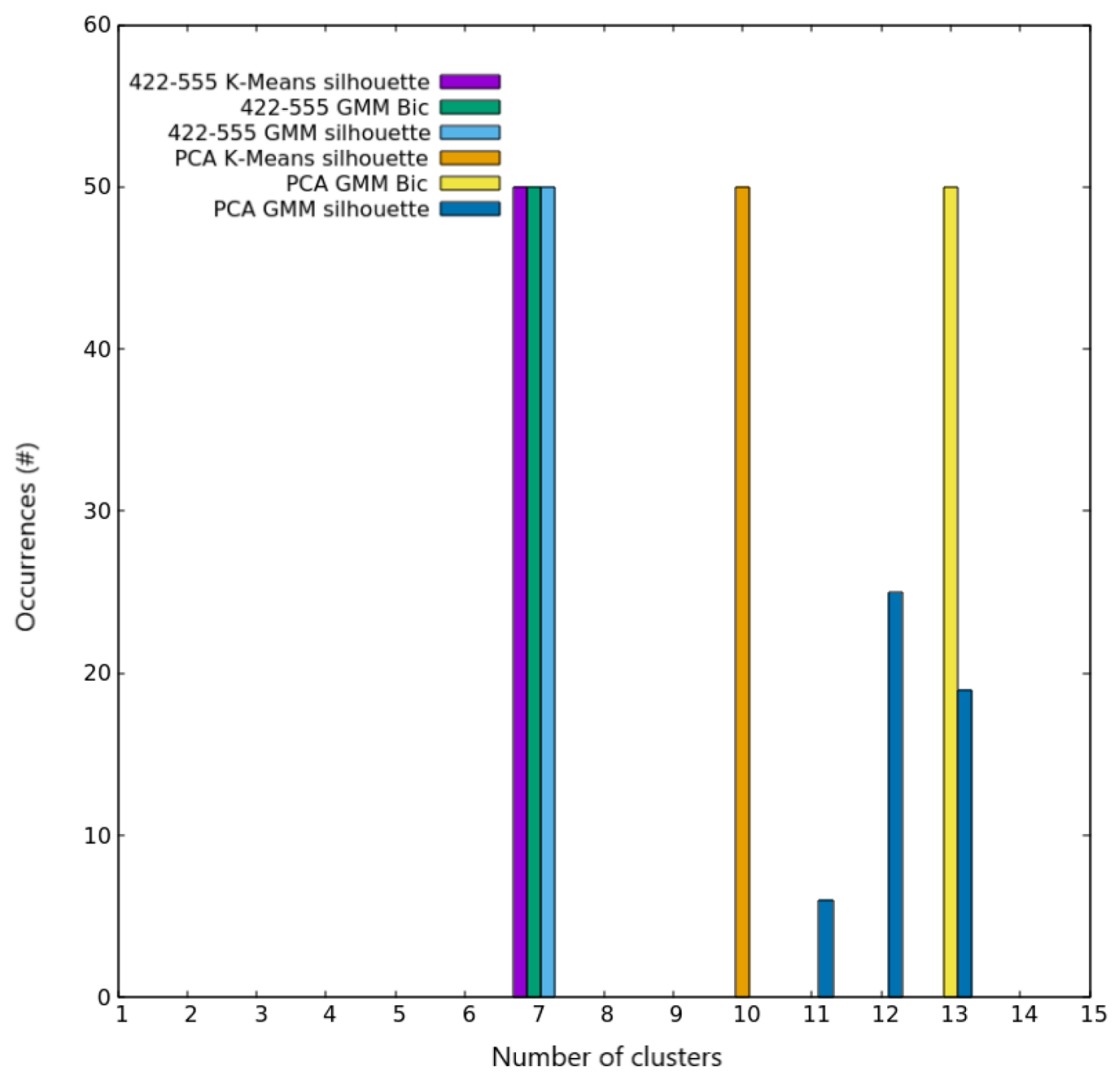
Clustering scores for AgCu nanoalloys with *N* = 100. Occurrences of the best number of clusters out of the 50 independent trials, as given by silhouette and Bic scores for both K-Means and GMM algorithms on the two different spaces used for describing nanoparticles.

**Figure 6:**
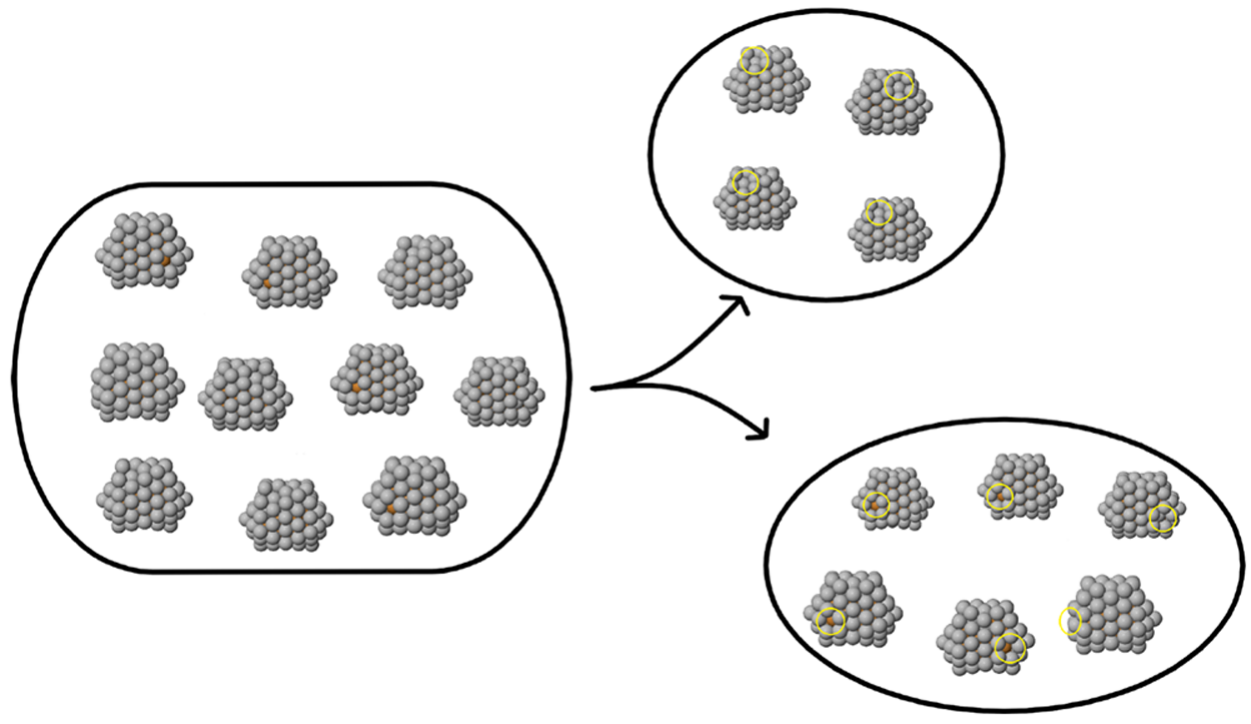
Subdivision of the *N* = 100 AgCu cluster of polyicosahedral structures. The two clusters on the right only differ for the displacement of one surface atom. In particular, the four nanoparticles in the top cluster miss an atom in the vertex position in their higher part, whereas the six nanoparticles in the bottom cluster also miss an atom in the vertex position but in their lower part. These missing-atom positions are highlighted by yellow circles.

We first considered the data set of ref (11), where AgCu nanoalloys with sizes *N* = 100 and *N* = 200 were globally optimized for different compositions. This set consists of *n* = 101 structures for both sizes. The results for AgCu nanoalloys with *N* = 100 already published in that reference are recovered with some modifications. In particular, the seven clusters found there were again found as the optimal separation by both K-Means and GMM on the 422–555 space, as shown in Figure 5. The implementation of the 64-dimensional description of these nanoalloys and the subsequent application of PCA led us to use the first four principal components, explaining 94.8% of the variance, since the first three explained 89.4%. In this space, the two algorithms found a higher number of clusters as the best possible separation of data. In particular, the best silhouette score for K-Means was found to be *k* = 10 in all cases (see again Figure 5), whereas for GMM the best number of clusters was found to be *k* = 13 and *k* = 12 for the Bic and silhouette score respectively, as it can be seen in the same figure. Manual inspection revealed that these clusters were obtained by splitting into smaller clusters some of the seven previously found. An example of this cluster subdivision can be seen in Figure 6, where the *N* = 100 AgCu polyicosahedral cluster (labeled as cluster (5) in section 3.1 of ref (11)) is split into two subclusters that differ in the displacement of one surface atom only.

**Figure 7:**
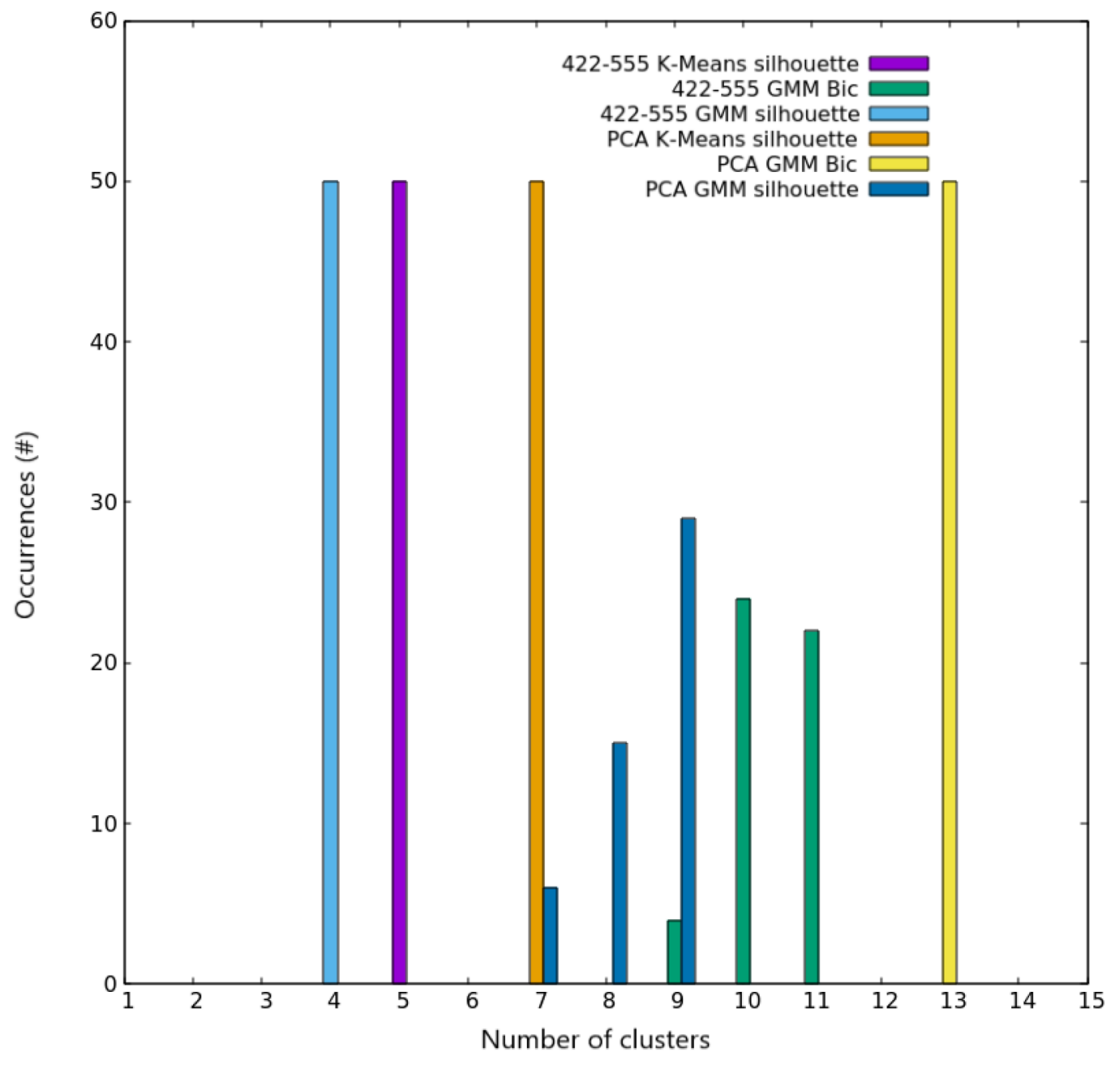
Clustering scores for AgCu nanoalloys with *N* = 200. Occurrences of the best number of clusters out of the 50 independent trials, as given by silhouette and Bic scores for both K-Means and GMM algorithms on the two different spaces used for describing nanoparticles. Colors are explained in the inset.

Similarly, the five clusters found in the same reference for *N* = 200 are again recovered with eventually some further splitting. Using the 422–555 space, the silhouette scores of K-Means and GMM point out *k* = 5 and *k* = 4, respectively, as the optimal number of clusters (see Figure 7), whereas the Bic score of GMM outputs more frequently *k* = 10 as the optimal number of clusters. These clusters are split even further by the application of GMM on the three-dimensional space given by PCA, which in this case explains 90.7% of the variance of the data set. Here, the Bic score is minimized unambiguously for *k* = 13, as shown again in Figure 7. A smaller number of clusters is instead found by the application of the silhouette score both for K-Means and GMM, giving *k* = 7 and *k* = 9, respectively, as shown in the same figure.

### AuPd, *N* = 250

**Figure 8:**
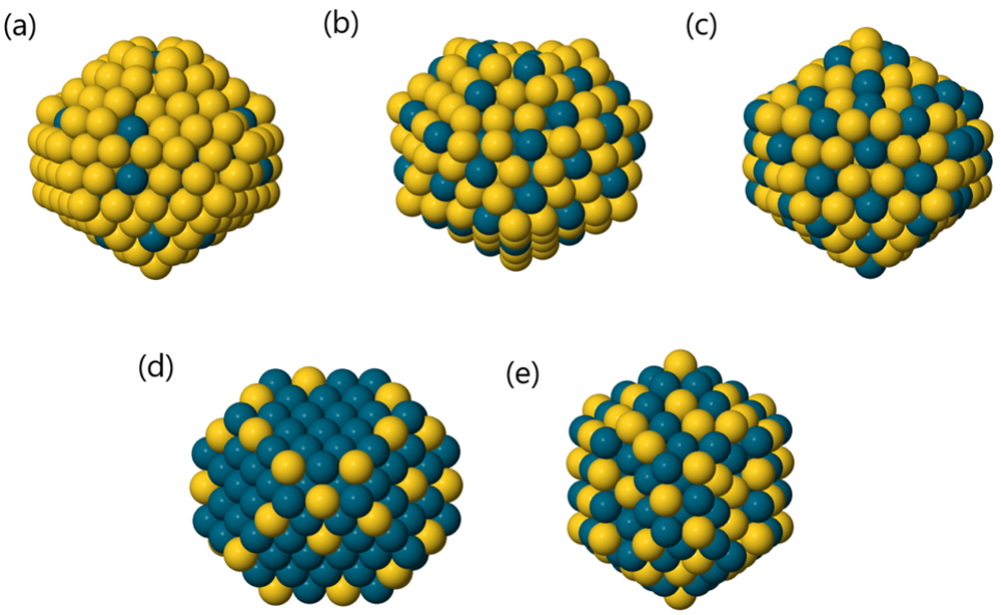
Five different structural motifs for AuPd nanoalloy global minima with *N* = 250: (a) first icosahedral motif, (b) second icosahedral motif, (c) third icosahedral motif, (d) fcc motif, and (e) decahedral motif.

From data sets of ref (23), a total number of 84 structures obtained from the global optimization of AuPd nanoalloys at size *N* = 250 and different compositions were collected. In our previous work,^23^ this data set was manually divided into five different groups: fcc, Dh, and three types of Ih, corresponding to different surface reconstructions (see Figure 8).

**Figure 9:**
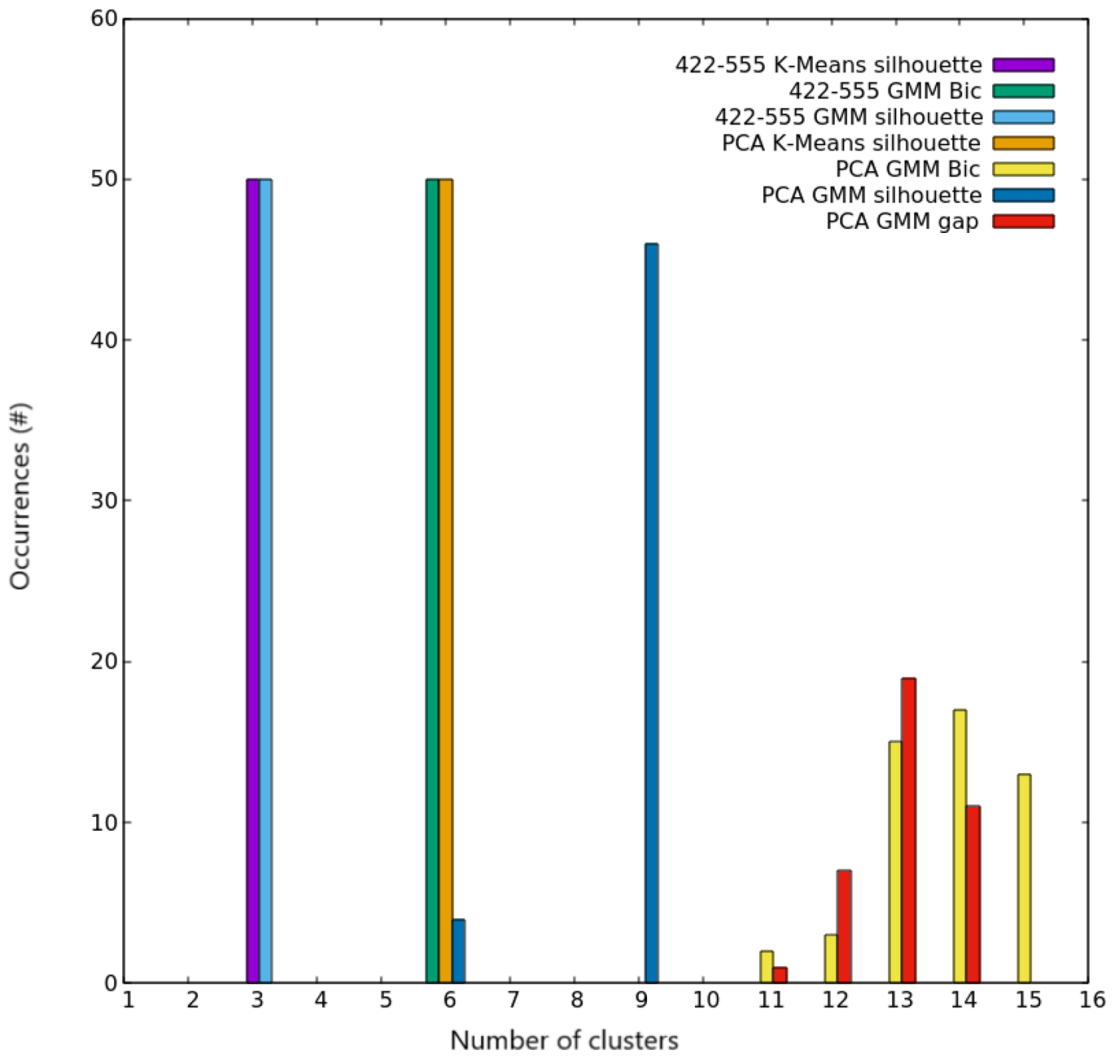
Clustering scores for AuPd nanoalloys with *N* = 250. Occurrences of the best number of clusters out of the 50 independent trials, as given by silhouette, Bic, and gap scores for both K-Means and GMM algorithms on the two different spaces used for describing nanoparticles. Colors are explained in the inset.

Using the two-dimensional description of 555 and 422 signature order parameters, we found that K-Means and GMM silhouette scores are maximized for *k* = 3 (Figure 9), whereas the Bic score calculated after the GMM fit is minimized when *k* = 6. When *k* = 3, the clusters found show Dh, fcc, and Ih motifs. On the other hand, when *k* = 6, the whole icosahedral cluster is split into four subclusters. In particular, the three icosahedral groups represented in Figure 8(a,b,c), i.e. those that had already been discovered manually, are recovered; moreover, the first one is split into two groups.

When the 64-dimensional description is implemented and clustering is performed after PCA, which explains 98.7% of the variance of the entire data set, different results are obtained. The silhouette score related to the K-Means fit dictates that the optimal number of clusters should be again *k* = 6 (see again Figure 9). This separation into six clusters on this space is very similar (almost identical) to that found by GMM on the 422–555 signature order parameter space. However, when GMM is implemented, the Bic score points out *k* = 14 as the optimal number of clusters (Figure 9, yellow bars), whereas the silhouette score is maximized most of the time for *k* = 9 (Figure 9, blue bars); the gap statistic prefers instead *k* = 13 (Figure 9, red bars). This result can be regarded as an example of some sort of overfitting (We discuss more deeply the meaning of this in the next section.). For instance, the 13 groups found by GMM are obtained by dividing into smaller parts the first and second icosahedral groups. However, the difference between these clusters is so little that it is almost unnoticeable. This does not mean that the clustering result is wrong, rather it is too precise for our purposes. A possible reason for this is that when dealing with such data sets, which have a lot of identical structures, and the description is really fine (even if the original 64-dimensional space has been mapped in a three-dimensional one that preserves its variance), then little departures from tightly bound clusters have a good chance to be considered as clusters themselves.

These two first systems showed that for nanoalloy structures obtained after global minimizations, it is possible to use different clustering approaches to perform a good separation into distinct geometrical families. It is shown in our results that here there are no wrong clustering choices. Rather, different descriptions or scores can lead to a more refined partition of the initial ensemble, resulting in a higher number of clusters found. We would like to remark that such a possibility is a consequence of the nature of the data set and also of the algorithms/scores employed. As long as there are no wrong intersections between clusters, such refined solutions should not be considered as a drawback of the scheme since they only offer a more detailed version of a result that can be already obtained with a coarser description and less flexible algorithms.

In the next subsections, we describe the results obtained for systems in which simpler methods do not give satisfying results anymore, and in which the new method that we propose is instead capable of finding a good clustering solution.

### Au, *N* = 309 and *N* = 315

**Figure 10:**
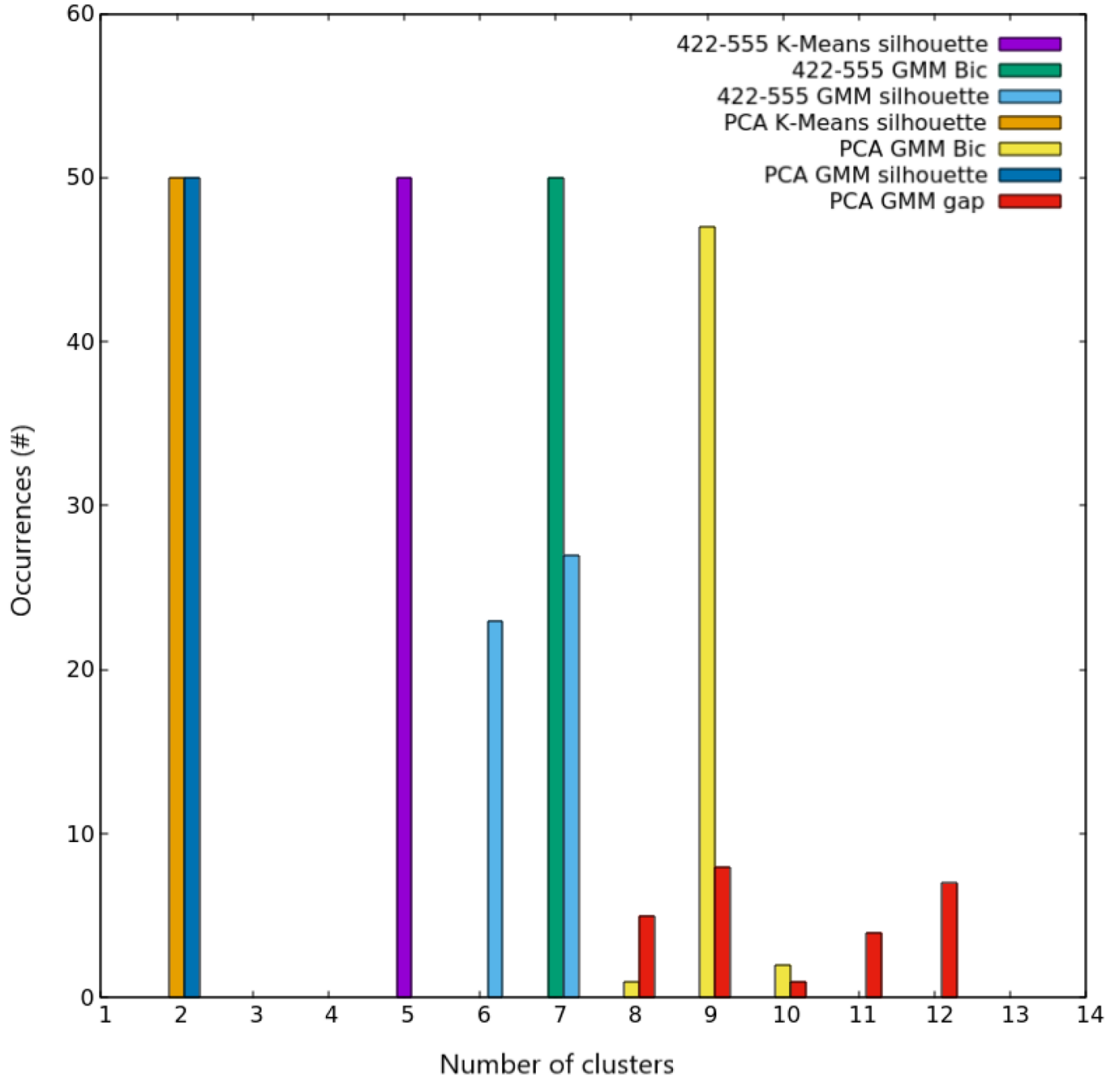
Au_309_ clustering scores. Occurrences of the best number of clusters out of the 50 independent trials, as given by silhouette, Bic, and gap scores for both K-Means and GMM algorithms on the two different spaces used for describing nanoparticles. Colors are explained in the inset.

We considered a data set composed of about six hundred structures sampled from a molecular dynamics trajectory for Au_309_ at 590 K. By implementing PCA, we discover that the three principal components explain 99.3% of the entire variance of the set. As anticipated, the combination of K-Means and silhouette score is not good enough to cluster NPs in this case, since the suggested number of clusters is *k* = 2 for all trials (see Figure 10), which is wrong. Instead, when the GMM is fitted, the Bic score dictates that the optimal number of clusters is *k* = 9, see Figure 10, and the same number is also singled out by the gap statistic.

**Figure 11:**
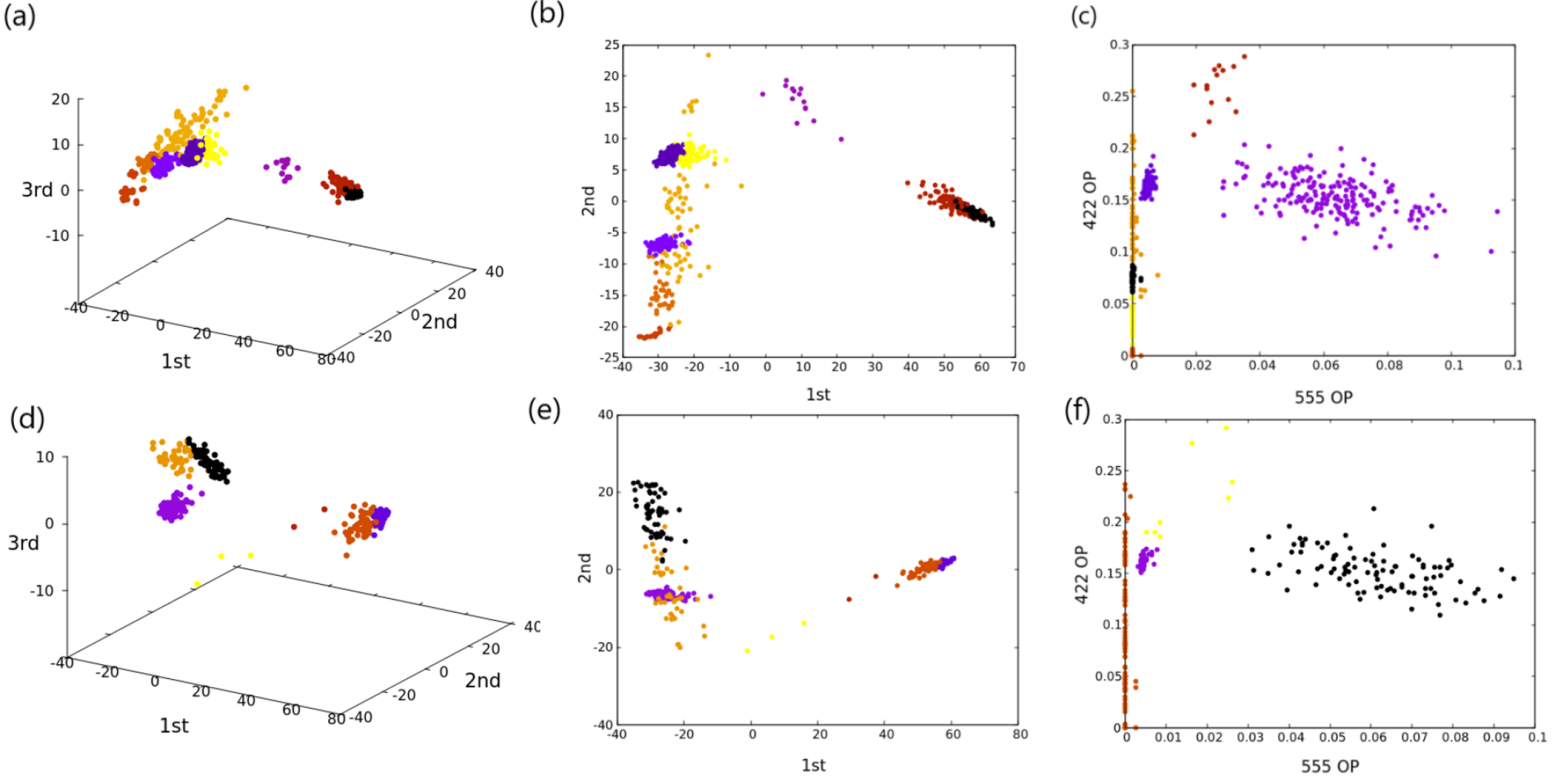
(a) *k* = 9 clusters for Au_309_ obtained with GMM in the three-dimensional space given by PCA. All axes are labeled with the corresponding principal component. Different colors refer to different clusters. (b) Projection of the same data along the first two principal components. (c) *k* = 7 clusters for the same data set obtained with GMM in the two-dimensional space given by 422 and 555 signature order parameters. (d) *k* = 7 clusters for Au_315_ obtained with GMM in the three-dimensional space given by PCA. All axes are labeled with the corresponding principal component. Different colors refer to different clusters. (e) Projection of the same data along the first two principal components. (f) *k* = 4 clusters for the same data set obtained with GMM in the two-dimensional space given by 422 and 555 signature order parameters.

The result of such separation can be seen in Figure 11(a,b), where points in the three-dimensional space given by PCA are colored differently for each cluster.

Manual inspection of the nine clusters reveal that, in most cases (In general, since any clustering algorithm solution may not be perfect, one should always consider a chance to get some structures belonging to the wrong structural family.), they contain the following structures:

- twin structures.
- fcc fragments with two ore more stacking fault islands or defected/faulted/asymmetric twin structures. This cluster is the most spread out among others, thus it is the one characterized by a higher degree of diversity between its elements.
- icosahedral fragments.
- decahedra.
- fcc fragments with one or two stacking fault islands. Also this cluster is quite blurred, revealing why it contains both structures with one or two stacking fault islands.
- fcc fragments with no stacking faults.
- decahedra with some surface defects or small disordered regions.
- liquid/amorphous structures.
- liquid/amorphous structures with a higher percentage of not-classified atoms.

**Figure 12:**
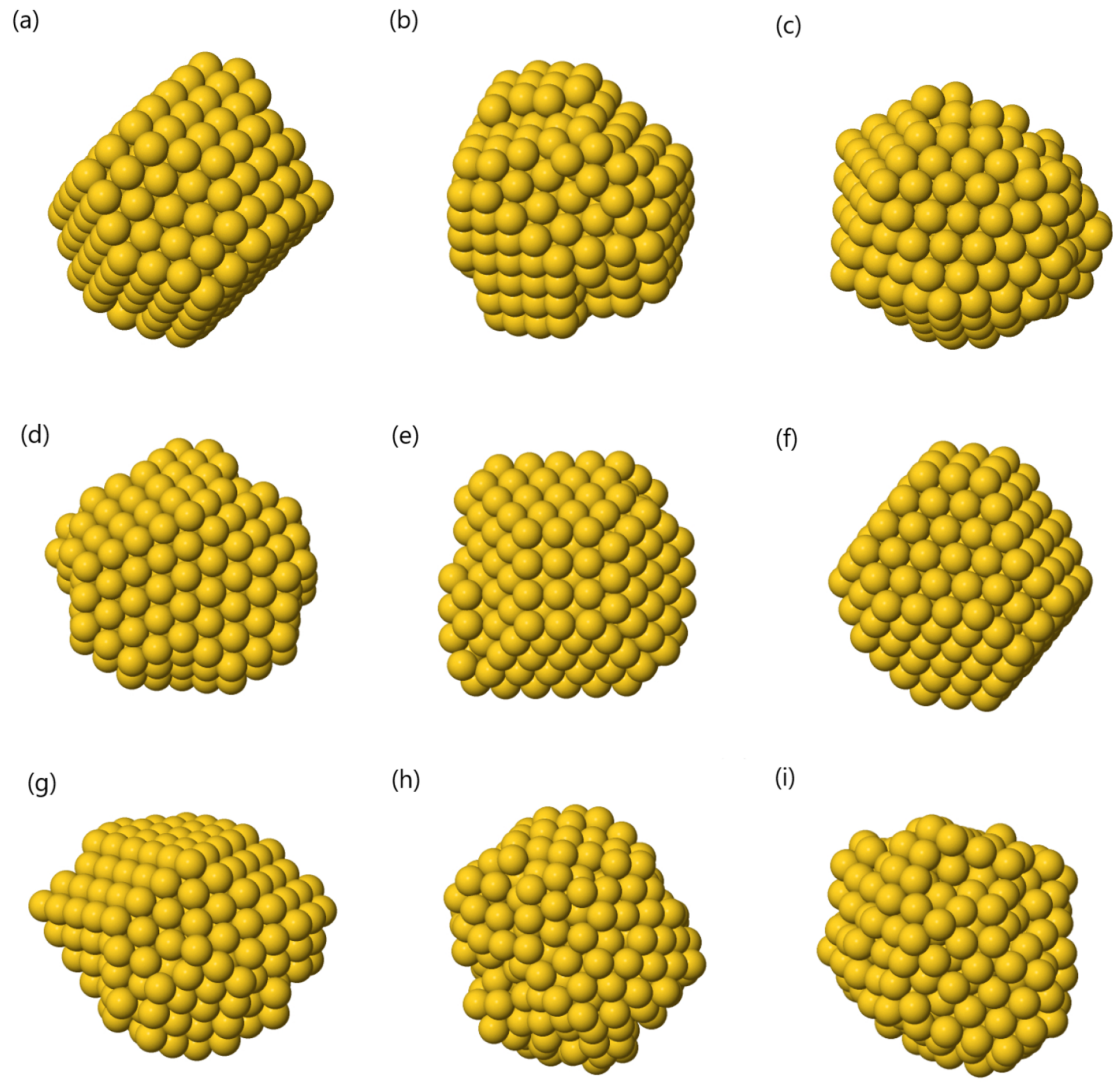
Au_309_ representative structures for the *k* = 9 clusters found by GMM in the three-dimensional space given by PCA, together with the Bic score: (a) twin structure, (b) fcc fragment with many stacking fault islands, (c) icosahedral fragment, (d) decahedron, (e) fcc fragment with a few faults, (f) fcc fragment with no faults, (g) decahedron with a disordered region on the surface, (h) liquid structure, and (i) another liquid structure.

A representative structure for each cluster can be found in Figure 12.

When GMM is fitted instead on the two-dimensional space given by 422 and 555 signature order parameters, the optimal number of clusters which minimizes the Bic score and maximizes the silhouette score is *k* = 7 (see Figure 10). These seven clusters are equal to those previously found, except for the two decahedral clusters which are merged into a single one as well as the two liquid clusters. On the same space, K-Means finds an optimal separation of data into *k* = 5 clusters; however, this tessellation leads to a wrong intersection of different structural families (such as Dh with fcc, liquid with Ih, etc.), and therefore it is discarded.

These results show that in this case there is only a little gain in using the three principal components of the 64-dimensional CNA space, since a good clustering result is already achieved by using GMM on the two-dimensional space given by 422 and 555 signature order parameters. However, the same conclusion does not hold for the case *N* = 315, as will be shown below.

**Figure 13:**
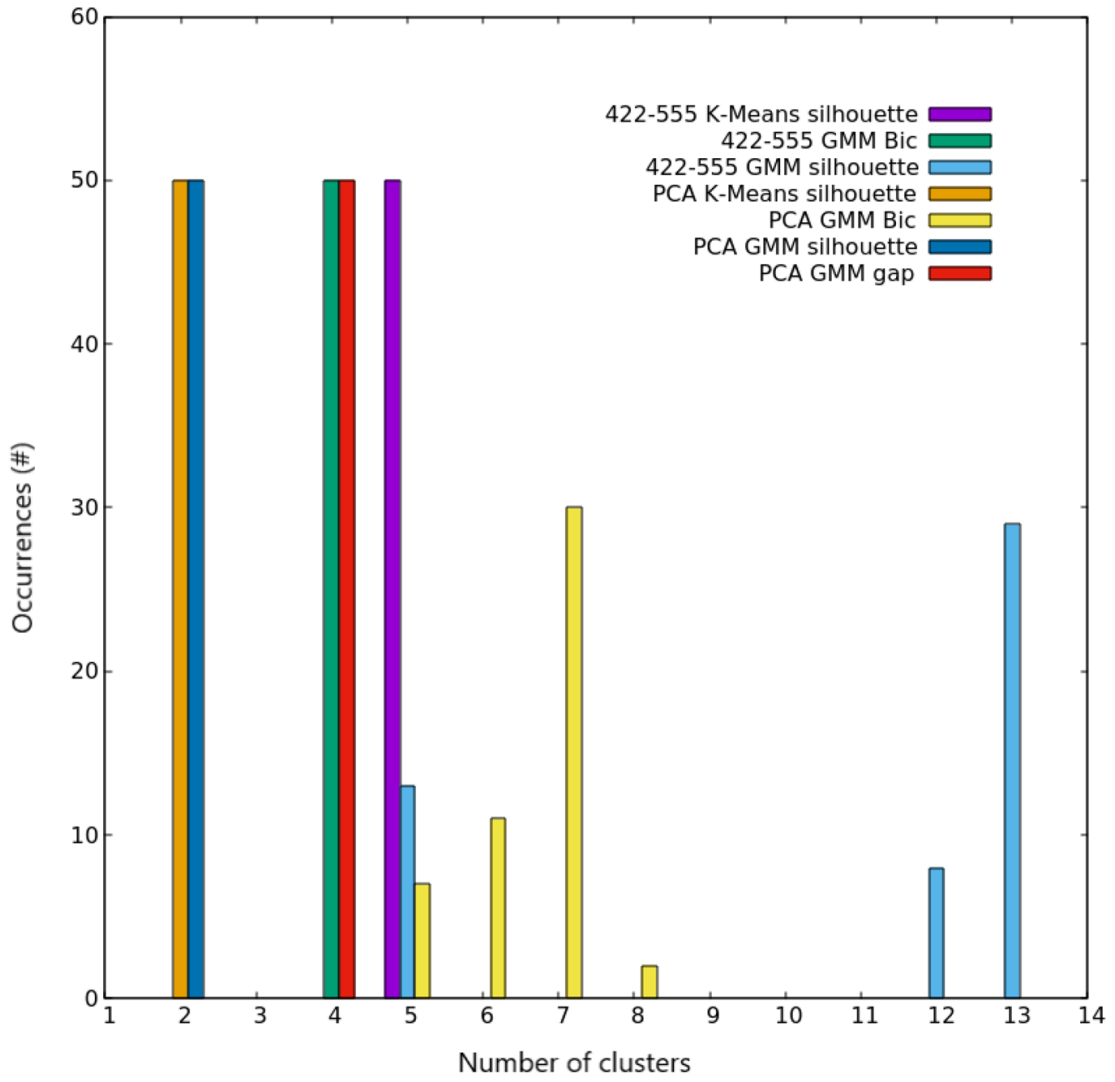
Au_315_ clustering scores. Occurrences of the best number of clusters out of the 50 independent trials, as given by silhouette, Bic, and gap scores for both K-Means and GMM algorithms on the two different spaces used for describing nanoparticles. Colors are explained in the inset.

In this case, *n* = 300 structures from a Au_315_ MD simulation at 590 K were analyzed with both methods. Here, the combination of PCA and GMM singled out seven different structural motifs (see Figure 13), including icosahedra, decahedra, liquid structures, and different kinds of fragments, with very similar structures as shown in Figure 12. Differently from before, the application of GMM in the 422–555 space was not as successful, finding four groups with some wrong intersections of structural motifs (see Figure 13) and Figure 11(f)). We note that in this case the gap statistic was not able to single out a good number of clusters for this system.

It is clear from these two examples that for such data sets, it is possible that a simple description of NPs as the one given by the two 422 and 555 order parameter signatures may not be good enough to allow the clustering algorithms to find a good separation of structures. Instead, the application of GMM after the dimensionality reduction given by PCA was good for both cases, as long as the Bic score was used.

### Ag, *N* = 147

**Figure 14:**
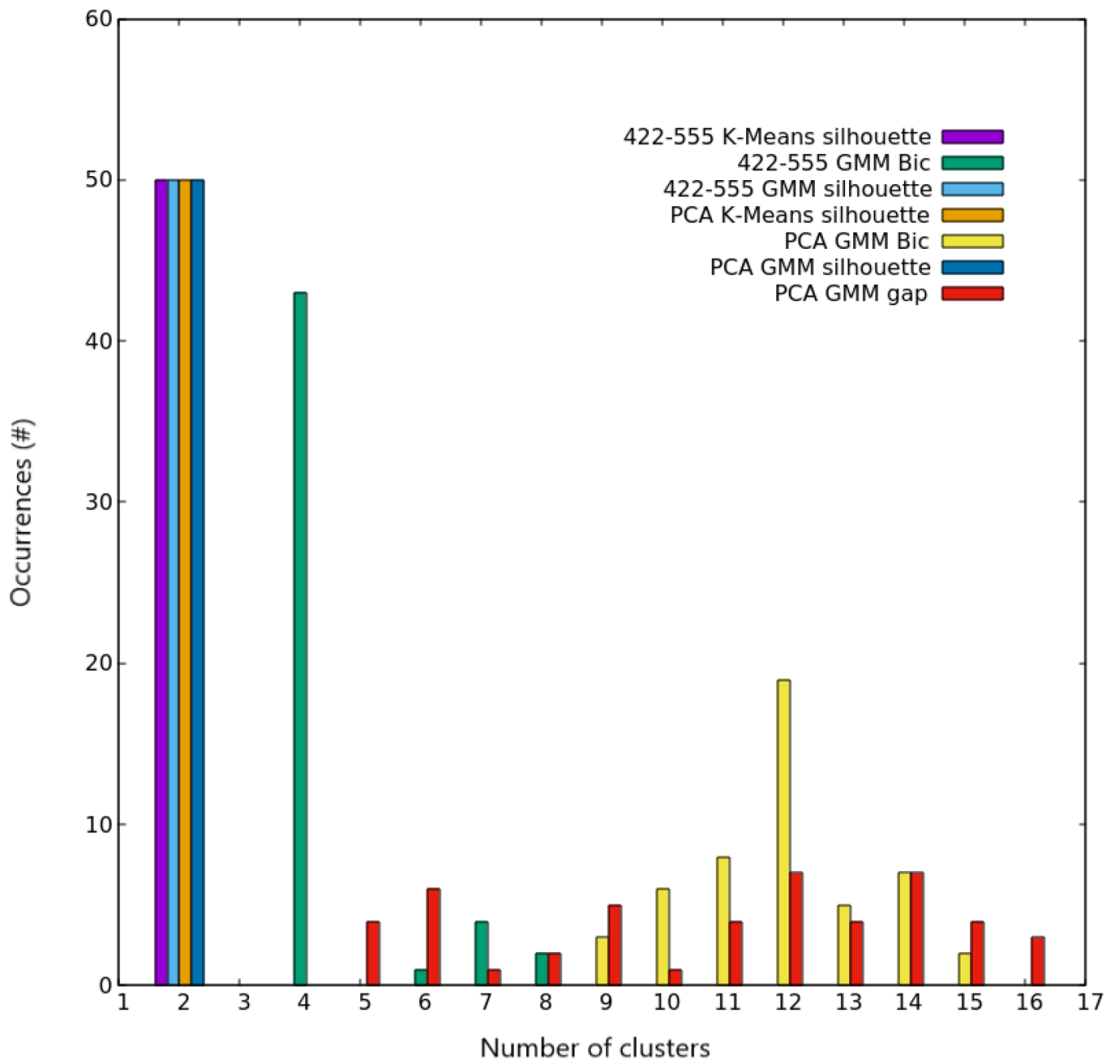
Ag_147_ clustering scores. Occurrences of the best number of clusters out of the 50 independent trials, as given by silhouette, Bic, and gap scores for both K-Means and GMM algorithms on the two different spaces used for describing nanoparticles. Colors are explained in the inset.

About three hundred structures from a Ag_147_ MD simulation at 650 K were given as an input to the two clustering approaches. The three principal components were able to explain 99.3% of the variance. In this case, the minimization of the Bic score for the PCA+GMM procedure dictated that the optimal number of clusters is *k* = 12 (see Figure 14), which is also preferred by the gap score, together with *k* = 14. We note that for this data set, both K-Means and GMM fail to find good clustering results on both spaces, when the silhouette score is used.

**Figure 15:**
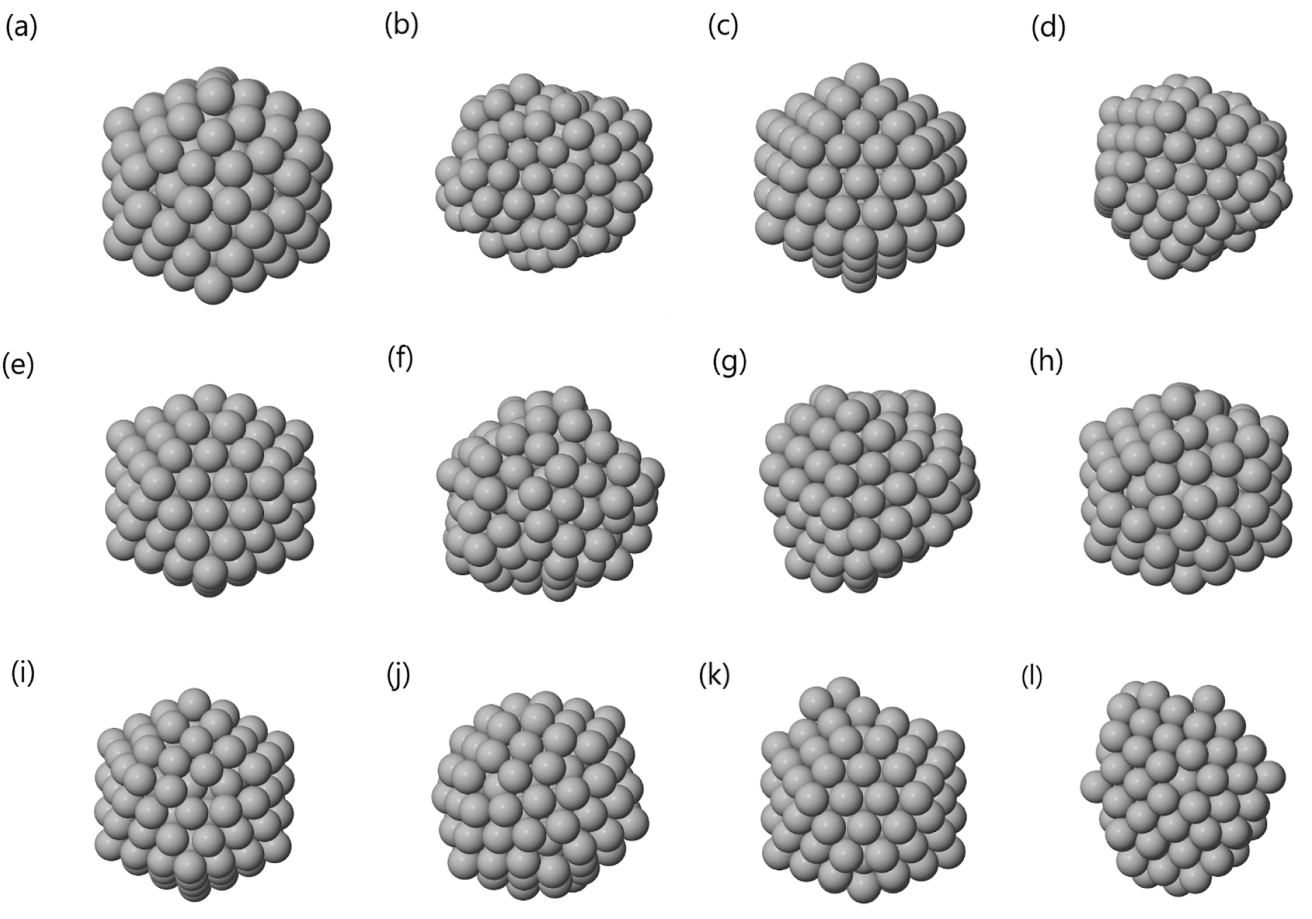
Ag_147_ representative structures for the *k* = 12 clusters found by GMM in the three-dimensional space given by PCA, following the indication of the Bic score: (a) icosahedron with rosettes, (b) liquid structure, (c) perfect Mackay icosahedron, (d) icosahedral fragment, (e) icosahedron without one vertex, (f) other liquid structure, (g) other kind of fragment, (h) icosahedron with distorted rosettes, (i) icosahedron with surface defects, (j) icosahedron without many vertexes, (k) icosahedron without two vertexes, and (l) decahedral fragment.

In this data set, there are no fcc fragments; among these 12 clusters, the algorithm was able to distinguish between liquid structures, different kinds of fragments, and some variations on an icosahedral theme. Among those variations there are icosahedra with different surface reconstructions, such as regular rosettes,^47^ distorted rosettes, surface defects, and one, two, or more than two missing vertexes. A representative structure for each cluster can be seen in Figure 15.

The application of GMM and Bic on the 422–555 space dictated instead that the optimal number of clusters should be *k* = 4. Manual inspection of these four clusters revealed that this procedure was able to single out liquid structures, fragments with also some liquid and icosahedral structures, and two groups of icosahedra, which contain all those already described above. Essentially, this result can be regarded as a coarser division of Ag_147_ NPs in a smaller number of groups, with again some wrong intersections between these groups.

Evidently, also in this case, the application of this procedure based on a more detailed description of NPs together with a reduction of the high-dimensional space was effective in detecting clusters thanks to the employment of GMM and Bic score.

### Au, *N* = 100

**Figure 16:**
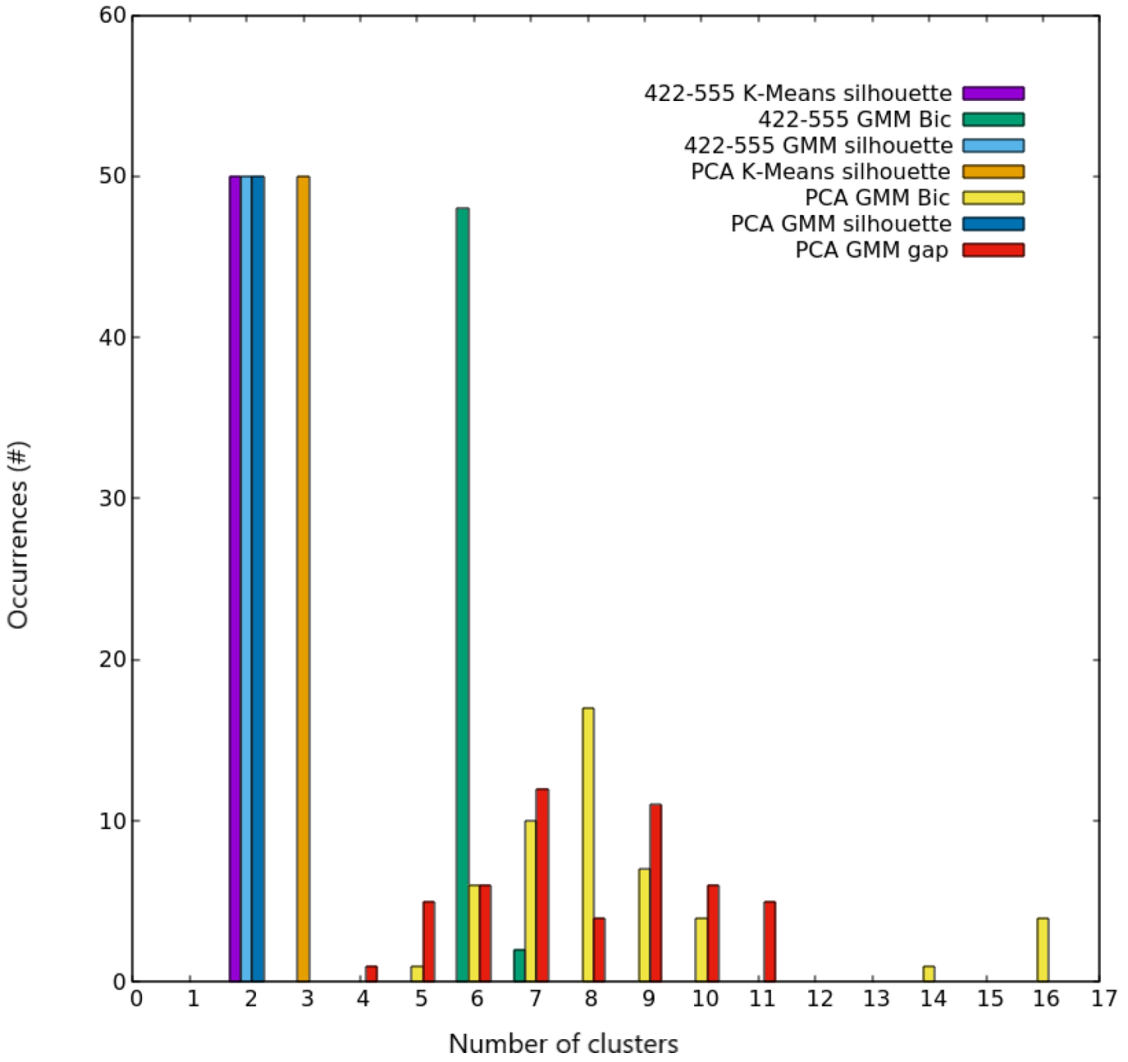
Au_100_ clustering scores. Occurrences of the best number of clusters out of the 50 independent trials, as given by silhouette, Bic, and gap scores for both K-Means and GMM algorithms on the two different spaces used for describing nanoparticles. Colors are explained in the inset.

Another challenging system to analyze is that of Au_100_ molecular dynamics at 420 K. At this temperature, which is just below the melting point for this size, the evolution of such a small system is very complicated since there are frequent fluctuations between isomers with comparable energy but very different and complicated structural motifs.^48^ Moreover, these fluctuations are not always stable, and some of these isomers only last a few nanoseconds. Here, the Gaussian Mixture Model on the three-dimensional space given by the principal components analysis minimizes the Bic score when *k* = 8, whereas the gap score prefers *k* = 7 (see Figure 16). The result is quite good, the algorithm being able to distinguish between decahedra, bidecahedra, fcc fragments, faulted fcc fragments, twin structures, and some polyicosahedra.

Also in this case, the K-Means algorithm is not able to discover a good separation of data on both spaces. Similarly as before, the GMM on the 422–555 finds out *k* = 6 clusters that are partially overlapped, due to the high presence of different isomers in this sample.

## Discussion

Through all the results that are presented here, a comparison between different choices of algorithms, variables, and scores was made in order to single out the most effective clustering methodology.

In general, we have experienced that data sets composed of structures obtained by global optimizations are well described both by the two-dimensional space given by the 422 and 555 signature order parameters and by the first principal components of the 64-dimensional space described above, meaning that in these spaces there is a good chance to obtain tightly bound clusters that are simple to identify. In these cases, K-Means and GMM are reliable tools to cluster the data set, and both silhouette and Bic scores can be used to evaluate the optimal number of structures. However, in global optimizations searches, there may be a tendency to produce very close points in such spaces or even identical points when, for example, two nanoalloys with different compositions share exactly the same geometrical shape and so the same vector of CNA signatures. In these cases, clustering algorithms overfit the data set (To be precise, in unsupervised learning, overfitting is not defined, but we try to clarify in the following.). When this is the case, the score outputs an optimal number of clusters which is greater than the “real” one, meaning that the precision by which clustering is carried out to divide the data set is too high even for being detected and/or appreciated at eye level. This scenario is realized for example when the Bic score has a high value in the log-likelihood term in eq 4, which, in turn, shifts the position of the minimum in the high range of *k*. Such a possibility explains why the word “overfitting” is not so out of context in this case. We note that this possibility should not be considered as a real drawback but rather as a opportunity to obtain a more refined grouping of structures.

On the other hand, structures obtained by MD simulations offer much more complex scenarios, since they usually involve fluctuations of structures and even amorphous/liquid-like NPs (especially if, for a given size, the simulation runs at a sufficiently high temperature). Moreover, due to the nature of physical trajectories, evolution within these structural motifs are followed with a certain degree of continuity. Thus, in the two above-mentioned spaces, structures are projected in much more elongated and blurred distributions, resulting in a much more difficult clustering problem, especially for smaller NP sizes (i.e., for higher surface to volume ratios). We found that, in these cases, K-Means usually fails to perform a good clustering and that even GMM often poorly performs on the 422 and 555 signature order parameter space, whereas it still gives good results on the PCA reduced space. For the same reason, also the silhouette score becomes less and less representative with an increase in elongation and blur, so that, if one has to resort to a single score, the Bic score becomes the most suitable choice.

## Conclusion

**Figure 17:**
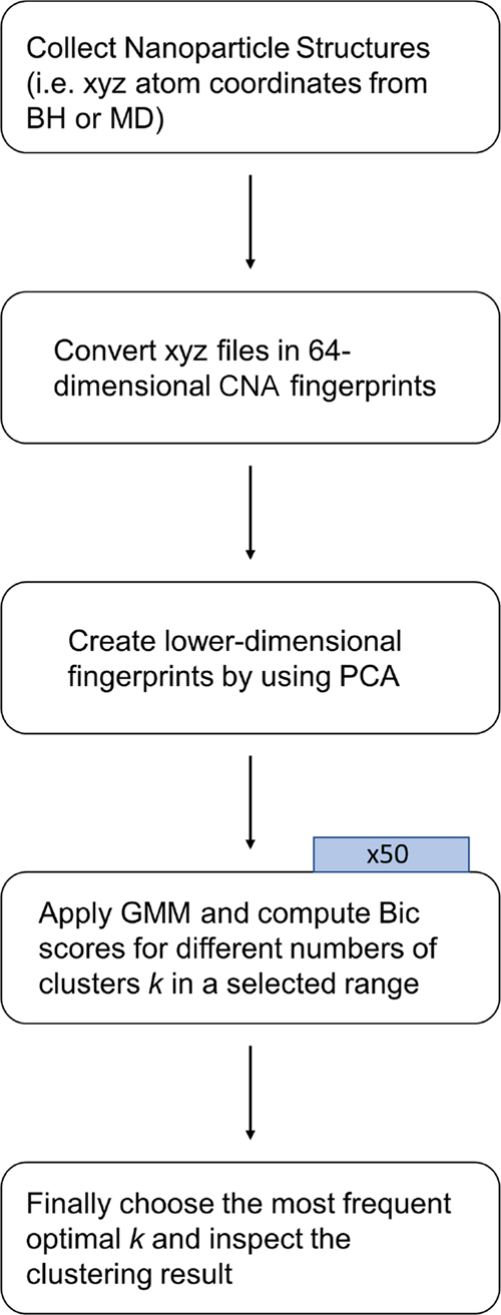
Our scheme workflow described in blocks.

In conclusion, we presented a scheme for clustering the structures of metal nanoparticles based on a combined approach of Common Neighbor Analysis and Machine Learning. By analyzing several examples of increasing structural complexity, we showed that the most effective strategy consists of three steps: (i) using a high-dimensional description of the local environment of each atom in the nanoparticle; (ii) reducing the dimensionality of the description by means of Principal Component Analysis (PCA); and (iii) clustering in the reduced space by virtue of the Gaussian mixture model (GMM) and assigning the scores by the Bayesian information criterion (Bic). The workflow of this scheme is represented in Figure 17.

We believe that this scheme could be used for different applications involving the problem of clustering nanoparticles given their atomic coordinates, thus acting as a starting point for the analysis of data sets of different complexities. The most evident application involving the problem of clustering nanoparticles is the analysis of a data set that includes different geometrical structures, which has served as the main motivation for our work. In practice, this is always the case for global optimization searches and molecular dynamics simulations. In fact, any MD simulation of interest has at least one atom diffusing inside the nanoparticle or on its surface, thus creating a variety of closely related structures (Otherwise if nothing happens - for example if the temperature is low - the nanoparticles always look the same, and there is no need for clustering.). Other types of simulations are for example those examined in this work, in which, at a sufficiently high temperature, nanoparticles are allowed to fluctuate between different isomers. Another type of simulation that was not considered here (but that would not change the substance of our work) is that of a solid–liquid transition, in which temperature is increased by a given rate. At some point, the nanoparticle undergoes a transition toward the liquid state, so that the analysis of the different structural types explored by the simulation could be of some interest. Finally, another application is that involving the calculation of probabilities for the different structural families as a function of temperature. This can be done for example by applying the Harmonic Superposition Approximation (HSA), which allows to compute the partition function of a nanoparticle in the canonical ensemble.^49^ From this computation one can go back to the probability of a particular structural family. The procedure, which was explained in detail and implemented in our previous work (see sections 2.4 and 3.4 of ref (11)), is the following: given a data set, one first applies the clustering scheme given in this work. By applying HSA, one then calculates the partition function (which will depend on temperature) for each nanoparticle of each cluster and also the sum of all these partition functions. The probability for one structure is then calculated by dividing its partition function by the sum of all partition functions. Finally, the probability of a particular cluster of nanoparticle structures as a function of temperature can be calculated just by taking the sum of the probabilities of nanoparticles belonging to that cluster.

## Data Availability

Nanoparticle structures (i.e., 3D atomic coordinates) were collected from global optimizations and molecular dynamics simulations, both by using our own code. The transformation from atomic coordinates to CNA coordinates in the 64-dimensional space was achieved again by using our own code. Application of Machine Learning algorithms (K-Means, GMM, PCA) and scores (silhouette, Bic) was done by using the open-source python module scikit-learn^32^ (version 1.0.2), available at https://scikit-learn.org/stable/, with an exception for the gap statistic, which was calculated using our own code. All xyz files of the nanoparticle structures as well as our scripts can be downloaded at the following Zenodo repository: https://zenodo.org/record/7085301.

## Supporting Information Available

Atomistic potential details and full list of CNA signature combinations (PDF)
