# Supplementary material for: Machine Learning Assisted Clustering of Nanoparticle Structures
Source: J Chem Inf Model. 2023 Jan 4;63(2):459–73. doi: 10.1021/acs.jcim.2c01203 (PMC9875306; doi:10.1021/acs.jcim.2c01203)
Supplement: Supplementary file 1 — ci2c01203_si_001.pdf [file ci2c01203_si_001.pdf]

Supporting Information

Machine Learning Assisted Clustering of Nanoparticles Structure

Cesare Roncaglia† and Riccardo Ferrando*,‡

†*Physics Department, University of Genoa, Via Dodecaneso 33, 16146 Genoa, Italy*

‡*Physics Department, University of Genoa, Via Dodecaneso 33, 16146 Genoa, Italy and CNR-IMEM*

E-mail: ferrando@fisica.unige.it

Atomistic potential

The potential energy of a nanoparticle can be written as a sum of single atomic contributions:

$$E = \sum_j E_j = \sum_j (E_j^b + E_j^r), \tag{1}$$

where

$$E_j^b = -\sqrt{\sum_{i \neq j} \xi_{sw}^2 \exp\left[-2q_{sw}\left(\frac{r_{ij}}{r_{sw}^0} - 1\right)\right]}, \tag{2}$$

is the negative binding term due to attractions, and

$$E_j^r = \sum_{i \neq j} A_{sw} \exp\left[-p_{sw}\left(\frac{r_{ij}}{r_{sw}^0} - 1\right)\right]. \tag{3}$$

models the positive repulsive term. Here r_{ij} is the distance between atoms i and j, and s (w) refers to the chemical species of the atom i (j). If $s = w$, r_{sw}^0 is the nearest-neighbour distance in the corresponding bulk lattice, while for $s \neq w$, r_{sw}^0 is taken as the arithmetic

mean of the distances of pure metals. Interactions between pairs of atoms are cutted-off in order to truncate the sums. In particular exponentials in Eqs. (2-3) are replaced by fifth-order polynomials, of the form $a_3(r - r_{c2})^3 + a_4(r - r_{c2})^4 + a_5(r - r_{c2})^5$, between distances r_{c1} and r_{c2} (which are second and third neighbour distances in the bulk lattice respectively), with a_3, a_4, a_5 fitted in each case to obtain a function which is always continuous, with first and second derivative for all distances, and goes to zero at r_{c2}.

CNA list

In Table S1 the list of all the 63 different combinations of signatures is given.

Table S1: List of our 63 possible atom classifications. In the first column named "NN" it is written the number of nearest neighbors of the classified atom.

NN	list of signatures	name
14	444 444 444 444 444 444 666 666 666 666 666 666 666 666	inner bcc
14	433 433 433 433 433 433 555 555 555 555 555 555 666 666	disclination polyIh
13	444 444 444 444 444 544 544 544 544 666 666 666 666	bcc subsurface 100
13	433 433 433 433 433 433 555 555 555 555 555 555 666	disclination polyIh end
12	421 421 421 421 421 421 421 421 421 421 421 421	inner fcc
12	421 421 421 421 421 421 422 422 422 422 422 422	inner hcp
12	555 555 555 555 555 555 555 555 555 555 555 555	inner central Ih
12	422 422 422 422 422 422 422 422 422 422 555 555	inner 5-axis
12	422 422 422 422 422 422 433 433 544 544 555 555	subsusrface Ih chiral type 1
12	421 421 421 421 422 422 422 422 433 433 544 544	subsusrface Ih chiral type 2
12	421 421 422 422 422 422 433 433 544 544 555 555	subsusrface Ih chiral type 3
12	300 300 311 311 422 422 433 433 544 544 555 555	central atom rosette without vertex
11	311 311 311 311 421 421 421 421 421 421 421	subsurface 110 & stepB 111 fcc
11	311 311 311 311 421 421 421 422 422 422 422	stepB 111 hcp
11	300 300 300 300 300 422 422 422 422 422 555	around central & noncentral in Ih
11	300 311 311 322 421 421 421 421 422 422 422	around noncentral hole in Ih
11	311 311 311 311 421 421 421 421 421 422 422	around noncentral hole in Ih
11	211 300 300 311 311 421 421 422 422 422 422	around noncentral hole in Ih
11	200 300 300 300 300 322 422 422 422 422 555	around noncentral hole in Ih
11	433 433 433 433 433 555 555 555 555 555 555	missing 5-fold vertex<br>am central atom
10	211 311 311 311 311 421 421 421 421 421	stepA 111 fcc
10	211 311 311 311 311 421 421 421 422 422	stepA 111 hcp
10	322 322 433 433 433 433 444 444 666 666	terrace 110 bcc
10	300 300 311 311 311 311 421 421 422 422	reentrance middle dh
10	311 311 311 311 422 422 422 422 433 433	edge fcc sharp between hcp islands
10	311 311 311 311 433 433 433 433 544 544	edge 111-111 chiral Ih
10	200 200 300 300 322 422 422 422 422 555	around multiholes in Ih
10	200 300 300 311 422 422 422 433 433 555	around multiholes in Ih
10	200 300 300 300 322 322 422 422 422 555	around multiholes in Ih

9	311 311 311 311 311 311 421 421 421	terrace 111 fcc & 0001 hcp
9	211 311 311 322 322 421 421 422 422	terrace hcp 10m11
9	211 211 211 211 444 544 544 544 544	terrace 100 bcc
9	311 311 311 311 322 421 433 433 544	edge 111-111 chiral Ih
9	311 311 322 322 433 433 433 544 555	edge 111-111 chiral Ih
9	322 322 322 433 433 433 555 555 555	polyIh around vertex
9	200 200 200 200 200 200 211 211 211	around multiholes in Ih
8	211 211 211 211 421 421 421 421	terrace 100 fcc
8	200 200 211 311 311 421 422 422	terrace hcp 10m11<br>& edge anti-Mackay
8	311 311 311 311 322 322 422 422	edge 111-111 twin
8	200 200 322 322 322 422 422 555	edge anti-Mackay close to vertex
8	311 311 311 311 322 322 422 422	edge 111-111 chiral close to vertex
8	322 322 322 322 433 433 444 666	edge 110-110 bcc
8	211 211 211 433 433 444 544 544	edge 110-100 bcc
8	322 322 322 322 433 433 555 555	polyIh side
7	200 200 311 311 311 311 421	edge 111-111 row 110 fcc<br>& 0001_10m11 hcp
7	211 211 211 311 311 421 421	edge 111-100 fcc & 0001_10m11 hcp
7	200 211 211 311 322 421 422	edge 10m11_10m11 hcp
7	200 211 211 311 322 421 422	edge 10m11_10m11 hcp
7	200 200 300 311 311 322 422	missing 5-fold vertex surrounding atoms
7	200 200 211 211 422 422 433	missing 5-fold vertex am surrounding atoms
7	200 200 300 311 311 322 422	vertex 111-111 reentrance dh
7	322 322 322 322 322 322 666	vertex 110-110 bcc wide & vertex rosette
7	211 211 322 433 433 444 544	vertex 110-100 bcc
6	211 211 311 311 311 311	edge 111-111 sharp fcc
6	100 100 211 211 422 422	edge 100-100 twin
6	200 211 211 311 311 421	vertex 111-100 fcc & 0001-10m11 hcp
6	322 322 322 322 322 555	vertex 111-111 5-fold Ih dh
5	322 322 322 322 444	vertex 110-110 bcc sharp
5	100 211 211 322 422	vertex 111-100 twin dh
5	200 200 211 311 311	vertex 111_111 fcc sharp-wide
4	200 200 200 200	vertex 111-111 fcc 4-fold
4	211 211 322 322	vertex 111-111 perimeter dh
3	211 211 211	vertex 111-111 fcc 3-fold
